# Comprehensive Characterization of Prognostic Long Noncoding RNAs in Osteosarcoma

**DOI:** 10.1155/2020/6725753

**Published:** 2020-08-24

**Authors:** Hua Gao, Yuanyuan Guo, Miaomiao Zhang, Zuqiang Yi

**Affiliations:** ^1^Department of Oncology, Henan Provincial Hospital, Zhengzhou Airport Economy Zone, Zhengzhou City, Henan Province, China 450000; ^2^Department of Pharmacy, The First Affiliated Hospital of Zhengzhou University, Zhengzhou City, Henan Province, China 450000; ^3^Department of Osteology, Henan Provincial Hospital, Zhengzhou City, Henan Province, China 450000

## Abstract

The molecular mechanism of osteosarcoma (OS) based on protein-coding genes has largely been studied in the past decades. However, much remains to be explored when it comes to the role that long noncoding RNAs (lncRNAs) play in the pathogenesis and progression of OS and how they are associated with OS metastasis. In the present study, we collected RNA-seq-based gene expression data of 82 OS samples from the Therapeutically Applicable Research To Generate Effective Treatments (TARGET) database, along with their clinical information. We found that 50 lncRNAs were significantly associated with patients' survival by univariable Cox regression model. Moreover, we built multivariable Cox regression model based on 7 lncRNAs and successfully stratified patients into two risk groups, which exhibited significantly different prognostic outcomes. Significantly enriched Gene Ontology (GO) terms and Kyoto Encyclopedia of Genes and Genomes (KEGG) pathways detected by differential expression analysis on DEGs between the two groups with different prognostic outcomes were both immune-related, indicating that such GO terms and pathways are critical for OS survival. Among the seven lncRNA signatures, *AC011442.1* was predicted to act as an oncogenic driver in OS by correlation analysis of copy number alteration (CNA) and lncRNA expression, and it was predicted to regulate AMPK and hedgehog signaling pathways. In summary, the identification of novel prognostic lncRNAs in OS could not only improved our understanding of the lncRNAs involved in OS tumorigenesis or progression but also assist the diagnosis and development of molecularly targeted therapies for OS, which in turn benefit patients' survival.

## 1. Introduction

Osteosarcoma (OS) is among the most prevalent malignancies in children and adolescents [1]. According to previous study, it takes up approximately 20% of all bone cancers, which also makes it one of the most common primary skeletal tumors [2, 3]. For example, according to the American Cancer Society, the estimated number of newly diagnosed cases of skeletal malignancies in 2017 would reach 3,260 in the United States, among them there would be roughly over 600 OS patients [4]. Unfortunately, over one-fifth of osteosarcoma patients exhibit lung metastasis at the time of diagnosis, which often results in unsatisfactory prognosis [5]. No significant improvement in 10-year overall survival of OS patients has been observed since the 1990s [6]. The magical effect of traditional tumor resection surgery and chemotherapy seems to encounter a bottleneck as they had once improved overall 10-year survival of OS from 30% to about 50% in the 1970s, and with the advances in molecular biology and related techniques, molecularly targeted therapies have since emerged as a new option in the management strategy of various cancers, including OS.

It is crucial for the development of molecularly targeted therapies to identify metastatic-related biomarkers and underlying mechanism in OS, in order to deliver a more accurate prognosis prediction and therapeutic decisions [7]. Long noncoding RNAs (lncRNAs) attract researchers' keen attention worldwide as they play a critical role through epigenetic, transcriptional, and posttranscriptional mechanisms in diverse biological processes, such as tumor initiation, growth, and metastasis [8]. Though lncRNAs are not to be translated into proteins, they can function as key regulators through interacting with miRNAs, mRNAs, and proteins [8]. Many lncRNAs are identified to exert oncogenic or tumor suppressor functions in OS, such as *ZEB1-AS1* [9], *SPRY4-IT1* [10], *BCAR4* [11], and *MFI2* [12]. For example, previous studies have reported that lncRNA DANCR could function as a competitive endogenous RNA in OS, thereby promoting ROCK1-mediated proliferation and metastasis [13]. CEBPA-AS1, an antisense RNA of CEBPA, has the capability of inhibiting proliferation and migration and promoting apoptosis in OS via Notch signaling [14]. These studies demonstrate that lncRNAs can regulate the progression, metastasis, and prognosis of OS [15].

In this study, RNA-seq data and clinical information of patients with osteosarcoma from the TARGET database were processed with univariable Cox regression and random forest algorithm, and we selected seven long noncoding RNAs (lncRNAs); all of them have the potential to affect the survival of osteosarcoma patients and to construct a prognosis risk model. Based on the stratification offered by our model, the corresponding biological differences among osteosarcoma patients and how these characteristics would result in varied prognostic outcomes were further explored and explained.

## 2. Materials and Methods

### 2.1. Data Resources

We downloaded RNA-seq-based gene expression data (TPM, transcript per million), somatic copy number alteration (SCNA) data, and clinical data of 82 corresponding osteosarcoma patients from the TARGET (Therapeutically Applicable Research to Generate Effective Treatments) database [16]. The segmented SCNA was annotated by Ensembl gene annotation v37.75 [17]. The SCNA status for each was called as gain or loss only if the log2 ratio (tumor/normal copy numbers) was more than 0.6 or less than -0.6. To meet the requirement for data analysis, we only collected 82 osteosarcoma samples with matched SCNA, gene expression, and clinical data.

### 2.2. Selection of lncRNAs in OS for Prognostic Risk Model Construction

First, based on 9 biotypes for lncRNAs (which were 3prime_overlapping_ncRNA, antisense, lincRNA, macro_lncRNA, non_coding, sense_intronic, sense_overlapping, bidirectional_promoter_lncRNA, and retained intron) in Ensembl, we obtained a total of 3,159 lncRNAs that exhibited TPM (transcript per million) > 0.1 in more than half of the samples. The expression status of lncRNAs were firstly classified into high and low expression, respectively, based on the median of the expression levels. Combined with the clinical information, univariable Cox regression analysis was then performed with package Survival v3.1-11 in R v3.6.3 to pick up lncRNAs significantly related to the survival of the patients (log-rank test, *P* < 0.05). Utilizing the random forest algorithm in R package randomForestSRC with default options, we evaluated ranked those lncRNAs and built a multivariable Cox model based on the top 20 prognostic lncRNAs. Subsequently, we only retained the prognostically insignificant lncRNAs in the initial multivariable Cox model (*P* > 0.05) and built the optimal multivariable Cox model based on these prognostic lncRNAs.

### 2.3. Model Construction for Evaluating Osteosarcoma Prognostic Risk

Taking into consideration the expression of qualified lncRNAs in each patient and the patient's survival status, we applied multivariable Cox regression with survival package in R v3.6.3 to build our osteosarcoma prognosis risk model, and lncRNAs with significant contribution to the model were selected. These lncRNAs were used to construct a risk-scoring method, which assigned a score that reflected the risk of death to each osteosarcoma patient. The patients were then divided by the median score into the high-risk and low-risk groups, accordingly. We visualized the survival curves of the two groups of patients by the Kaplan-Meier method and assessed the differences between the two groups by log-rank test.

### 2.4. Functional Enrichment Analysis of the Dysregulated Genes in the Two Risk Groups

As osteosarcoma patients were categorized, their gene expression profiles fell into two groups, accordingly. Utilizing the screening criteria of ∣log2 (fold change) | >1 and *P* value < 0.05, genes with significant differential expression between the two groups were selected. Subsequently, Gene Ontology (GO) [18] and Kyoto Encyclopedia of Genes and Genomes (KEGG) pathway [19] enrichment analysis were performed on identified differentially expressed genes with the package clusterProfiler v3.12.0 in R v3.6.3.

### 2.5. Estimation of Immune Cell Infiltrating Levels

The infiltrating levels of immune cells were estimated based on the gene expression profiles and marker genes of immune cells. Single-sample gene set enrichment analysis (ssGSEA) was employed in this study. This analysis was implemented in R GSVA v1.32.0 package [20].

## 3. Results

### 3.1. Identification of Prognostic lncRNAs in OS

**Figure 1:**
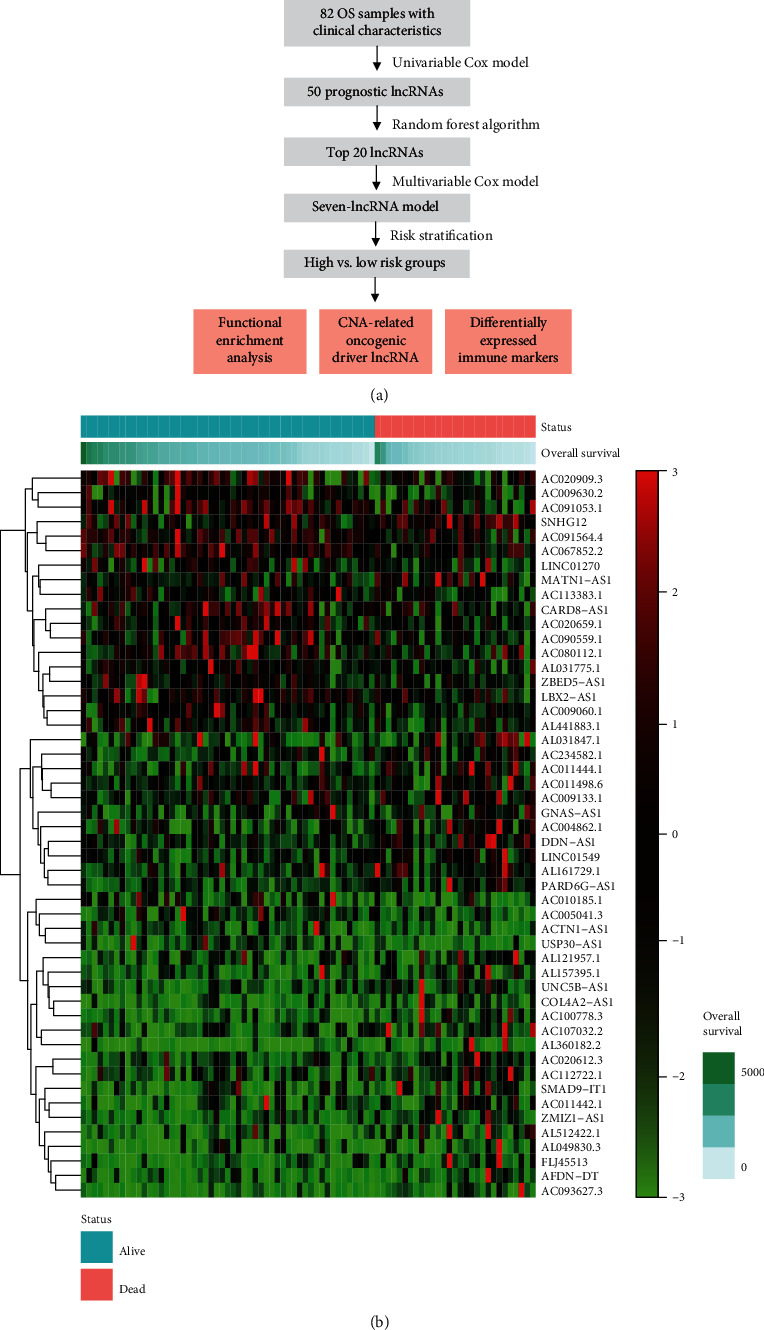
The study design and the expression profiles of the 50 prognostic lncRNAs in OS. (a) The workflow of the present study. (b) The lncRNAs were clustered by hierarchical clustering algorithm, and the samples were ordered by survival status and survival time.

As shown in Figure 1(a), the present study conducted a series of data analysis to build a predictive model for OS risk. The gene expression and clinical information of 84 osteosarcoma patients were obtained using the TARGET database, among which, two samples were excluded due to a lack of overall survival time. Based on the gene annotation from the Ensembl database and criteria regarding TPM, we selected 3,159 long noncoding RNA for later establishment of the prognostic risk model (see Materials and Methods). Among these 3,159 lncRNAs, we identified 50 lncRNAs significantly associated with patients' overall survival by univariable Cox regression model (log-rank test, *P* values < 0.05, Supplementary Table S1). As illustrated in Figure 1(b), the expression of the prognostic lncRNAs were significantly differentially expressed between the alive and deceased OS patients. These results indicated that the prognostic lncRNAs identified by the univariable Cox regression model may be essential for OS tumorigenesis and/or progression.

### 3.2. Construction of lncRNA-Based Multivariable Cox Model for Risk Prediction in OS Patients

**Figure 2:**
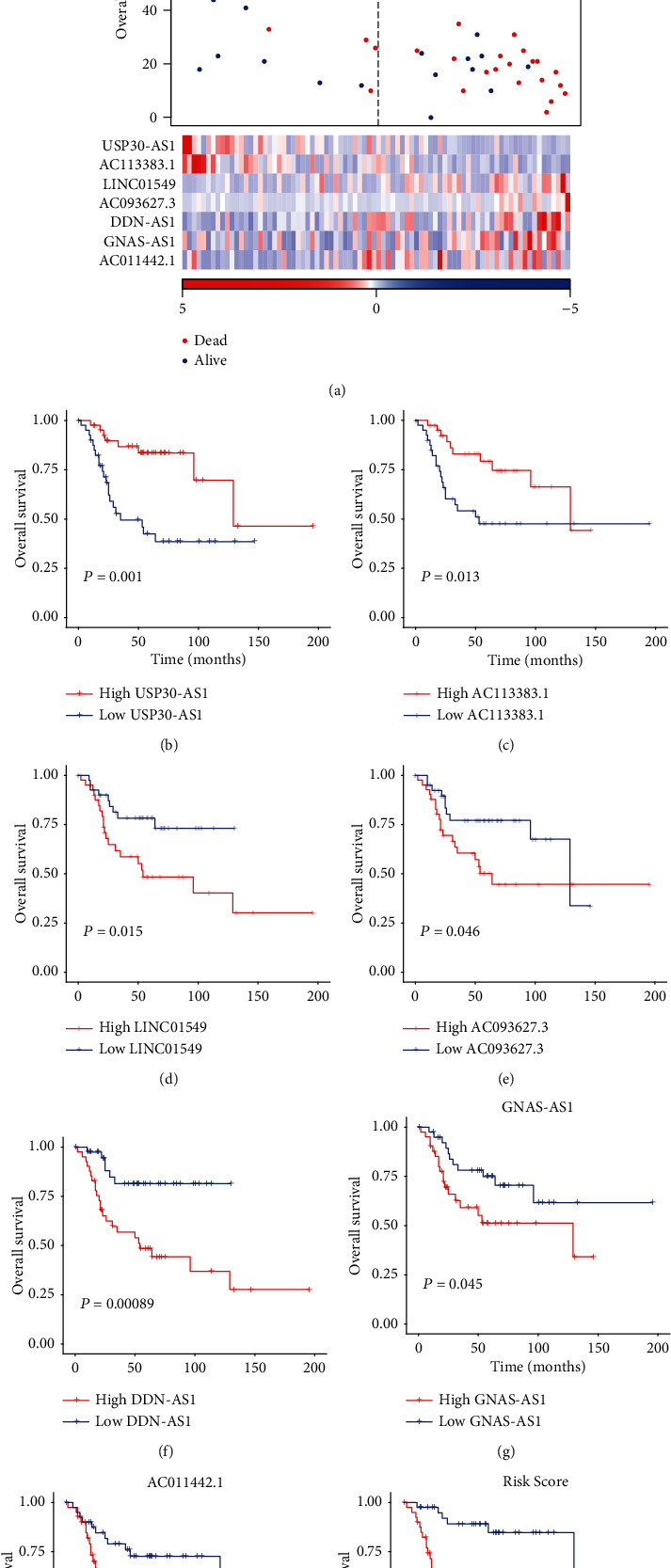
The performance of the seven lncRNAs in OS survival prediction. (a) Risk scores for each patient in different groups, where the blue points represent low-risk patients, and red points represent high-risk patients at the top panel. In the middle panel, the distribution of survival time and survival status of two groups of patients, of which the *y*-axis stands for survival time, blue points represent living patients, and red dots represent the dead patients. The expression patterns of the selected lncRNAs in each OS patient were displayed at the bottom. The Kaplan-Meier curves for survival of patients stratified by the seven lncRNAs were displayed in (b)–(h), respectively. (i) The Kaplan-Meier curve for the samples stratified by the risk score of multivariable Cox regression model.

To build a lncRNA-based Cox regression model for OS risk prediction, we first ranked the prognostic lncRNAs by random forest algorithm, and the top 20 lncRNAs were considered candidates for the construction of an OS prognostic risk model. We then built our model with multivariable Cox regression on the samples with clinic information and expression data of these lncRNAs and obtained seven lncRNAs that significantly contributed to the model (Table 1). Based on the multivariable Cox model, the OS patients were divided into two risk groups using the median risk score. As shown in Figure 2(a), the proportion of deceased samples in the high-risk group (high-risk) was much greater than that in the low-risk group (low-risk) (25/41 vs. 4/41, test of proportion, *P* < 0.05). Moreover, compared with the low-risk group, patients in the high-risk group exhibited significantly lower overall survival time (33.6 vs. 68.7, log-rank test, *P* = 1.54*E* − 5). Furthermore, patients were then divided into the high-/low-expression groups based on the expression profiles of these seven lncRNAs, respectively. The Kaplan-Meier curves showed a significant association of the seven lncRNAs with overall survival of patients with OS (Figures 2(b)–2(h)). Consistently, the risk score was observed to have a higher statistical significance than any of the seven prognostic lncRNAs (Figure 2(i)).

In addition, to assess the independence of this scoring in predicting patients' prognosis, we performed both univariable and multivariable Cox regression for samples using the precalculated risk scores and their clinical information such as gender, race, and age. We found that this risk score was an independent indicator for OS patients' survival (Table 2), further suggesting that the risk score by the seven-lncRNA-based Cox model had the potential to predict the risk of OS patients.

### 3.3. Functional Characterization of Dysregulated Genes in High-Risk and Low-Risk Groups

**Figure 3:**
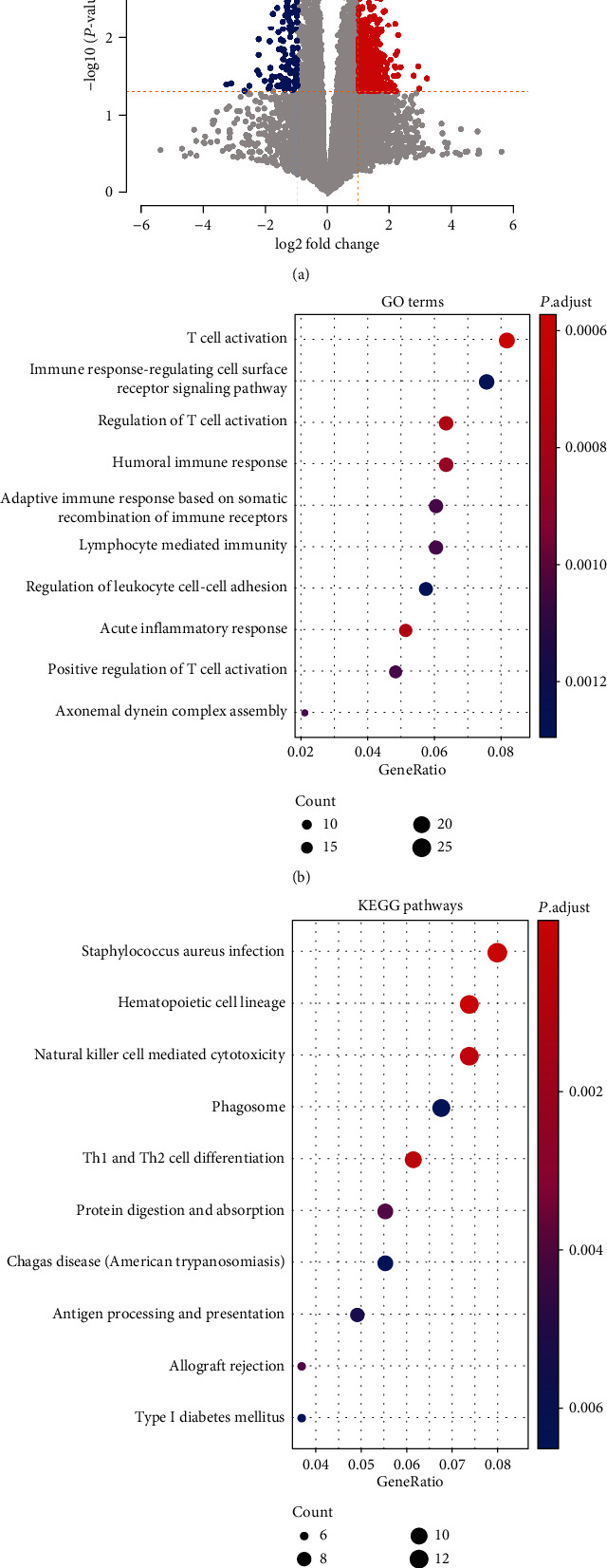
The biological differences between the high-risk and low-risk groups stratified by the multivariable Cox regression model. (a) The overview of the differentially expressed genes between the two risk groups. The red and blue points represent the upregulated and downregulated genes in the high-risk group compared with low-risk group. The differentially expressed genes were significantly enriched in GO terms (b) and KEGG pathways (c).

To investigate dysregulated genes in the two risk groups, we compared the gene expressions of these two risk groups. With thresholds at |log2 (fold change)| >1 and *P* value < 0.05, we identified 864 significant differentially expressed genes (DEGs), and when compared with the low-risk group, the expression of 728 gene was significantly upregulated in the high-risk group, and the expression of another 136 genes was downregulated (Figure 3(a)).

The GO and KEGG pathway enrichment analyses proved that the immune microenvironment of osteosarcoma patients played a crucial role in OS progression. It can be learned that the top 10 GO terms exhibited close association with immunity, including inflammatory responsive response T cell activation, humoral immune response, lymphocyte-mediated immunity, axonemal dynein complex assembly, positive regulation of T cell activation, and regulation of leukocyte cell-cell adhesion (Figure 3(b)), suggesting that the varied immune environment between the high- and low-risk groups may result in their prognostic differences. What is more, from the KEGG pathway enrichment analysis, we observed that a majority of the pathways, where these differentially expressed genes were significantly enriched, consisted of immune-related ones, such as NK cell-mediated cytotoxicity, staphylococcus aureus infection, Th1 and Th2 cell differentiation, antigen processing, and presentation (Figure 3(c)). The consistence between the GO and KEGG enrichment analyses further demonstrated the immune-related biological process may play a key role in OS progression.

### 3.4. AC011442.1 May Act as an Oncogenic Driver lncRNA in OS

**Figure 4:**
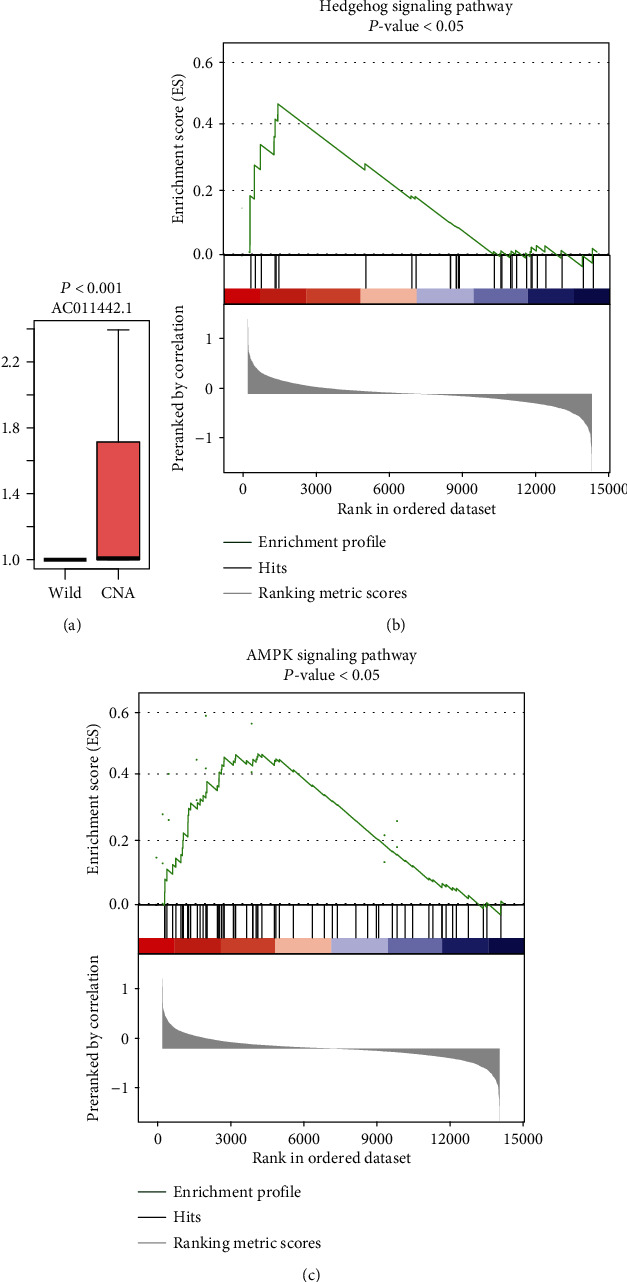
The oncogenic driver lncRNA AC011442.1 and its functionality. (a) The expression patterns of AC011442.1 in OS patients with CNAs and wild type. (b) The predicted pathways that the AC011442.1 might participate in.

As lncRNAs upregulated or downregulated by copy number alterations (CNA) probably acted as driver lncRNAs in cancer, we performed correlation analysis of the expression level and the corresponding copy number status for the seven prognostic lncRNAs in the multivariable Cox model. We observed that *AC011442.1* was highly upregulated in samples with CNA as compared with wild-type samples (*P* < 0.001, Figure 4(a)). Notably, the copy numbers of the four genes were frequently gained in OS samples (frequency > 10%).

To further investigate the biological function of the four lncRNAs, we conducted gene set enrichment analysis on protein-coding genes that highly correlated with identified lncRNAs. We found that *AC011442.1* was significantly and positively correlated with genes involved in the AMPK signaling pathway and hedgehog signaling pathway, respectively (Figures 4(b) and 4(c), *P* value < 0.05). These results indicated that *AC011442.1* may enhance the activities of the AMPK signaling pathway and hedgehog signaling pathway.

### 3.5. The Immune Markers Associated with OS Prognosis

**Figure 5:**
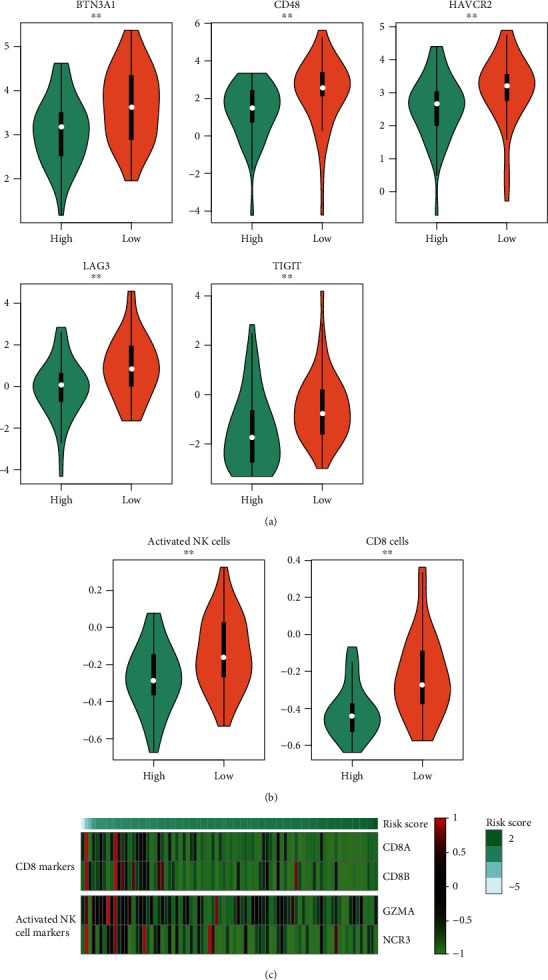
OS prognosis-related immune signatures. (a) The expression patterns of immune inhibitory receptors in the two risk groups. (b) The differential infiltrating levels of CD8 T and activated NK cells. The high-risk and low-risk groups are represented by the labels of “high” and “low” and colored by green and orange, respectively. (c) The expression profiles of the marker genes of the CD8 and NK cells. The samples are ordered by the risk scores.

To further explore the immune cells and related markers associated with OS prognosis, we first examined the expression patterns of the immune markers. Specifically, the immune inhibitory genes such as *BTN3A1*, *CD48*, *HAVCR2*, *LAG3*, and *TIGIT* were significantly upregulated in the low-risk group (Figure 5(a), *P* < 0.01), suggesting that the anticancer activity of the immune cells might be suppressed by these inhibitory genes. Furthermore, we also observed that the relative infiltrating levels of CD8 T cells and activated natural killer cells were attenuated in the high-risk group (Figure 5(b), *P* < 0.01), suggesting that the worse survival in the high-risk group of OS may be caused by the lack of CD8 and NK cells. Consistently, the marker genes of CD8 and NK cells, CD8A, CD8B, GZMA, and NCR3 were also downregulated in the high-risk group. These findings indicated that the immune cells and related markers were highly associated with OS prognosis.

## 4. Discussion

The molecular mechanism of OS based on protein-coding genes has largely been studied in the past decades. Despite extensive researches about the molecular mechanism of OS, there is still a lack of understanding of the lncRNAs' role in OS tumorigenesis, progression, and metastasis. Meanwhile, the identification of the prognostic lncRNAs involved in OS can facilitate the development of new diagnostic or therapeutic biomarkers.

In the present study, we collected 82 OS samples with RNA-seq-based gene expression data and their clinical information from the TARGET database. We found that 50 lncRNAs were significantly associated with patients' survival by a univariable Cox regression model (*P* values < 0.05). Through the selection of prognostic lncRNAs, we identified 7 lncRNAs with significant performance in OS survival prediction, built multivariable Cox regression model under the 7 lncRNAs, and successfully stratified patients into different risk groups with distinctive survival outcomes. Notably, *DDN-AS1*, one of the seven lncRNAs used by the multivariable Cox regression model, has been reported to act as competing endogenous RNA (ceRNA) that promoted the expression of TCF3 through competitively binding miR-15a and miR-16 [21], suggesting that *DDN-AS1* may promote OS progression in a similar manner. We further analyzed gene expression profiles of patients in different risk groups and obtained a list of DEGs. Functional enrichment analysis revealed that significantly enriched GO terms and pathways were associated with many aspects of immunity, indicating that immune-related functions are critical for OS survival, which is consistent with previous studies [22].

As dysregulated lncRNAs caused by copy number alterations (CNA) may act as driver lncRNAs in cancer, correlation analysis of the expression level and the corresponding copy number status for the seven prognostic lncRNAs was performed to identify the driver lncRNAs. Notably, *AC011442.1* was also predicted as one of the four-driver lncRNA (*P* < 0.001). Interestingly, *AC011442.1* was predicted to participate in cancer-related pathways [23–25], including the AMPK signaling pathway and hedgehog signaling pathway. Hedgehog signaling pathways have been frequently observed to drive tumorigenesis and metastasis of OS [26]. These results further demonstrated that the driver lncRNAs played a key role in OS, which could be used for further research of molecular mechanism.

As the exploration into varied molecular patterns between the two risk groups revealed that the immune-related pathways were enriched by DEGs in OS, we then examined whether the abundance of immune cells and markers were associated with OS prognosis. Specifically, the immune inhibitors such as *BTN3A1*, *CD48*, *HAVCR2*, *LAG3*, and *TIGIT*; CD8 T and activated NK cells; and related markers were significantly downregulated in the high-risk group. Particularly, CD48, HAVCR2, LAG3, and TIGIT were identified as novel immunotherapeutic targets of several cancers [27–30], suggesting that the low-risk OS patients might benefit from their candidate inhibitors.

In addition, the limitations of this study should be pointed out. Firstly, the multivariable Cox regression model needs an independent gene expression data for the validation of its robustness. Secondly, though a list of dysregulated lncRNAs associated with OS survival was identified, but future experimental verification is still needed. Moreover, detailed molecular functions of identified dysregulated lncRNAs had not been thoroughly discussed in this study. We hope that, when validation datasets become available in the near future, we can further confirm our findings and perform experimental validation. In summary, the identification of novel prognostic lncRNAs in OS would not only improve our understanding of the lncRNAs involved in OS tumorigenesis or progression but also assist the prediction of OS survival and development of molecularly targeted therapies to some extent, which in turn benefit patients' survival.

## Figures and Tables

**Table 1:** The summary for seven prognostic lncRNAs in univariable and multivariable Cox regression model.

Features	Univariable Cox regression			Multivariable Cox regression		
	Coefficient	Hazard ratio	*P* value	Coefficient	Hazard ratio	*P* value
USP30-AS1	-1.25	0.29	1.95*E* − 06	-2.10	0.122	4.22*E* − 03
AC113383.1	-0.09	0.91	8.34*E* − 03	-0.11	0.89	4.47*E* − 03
LINC01549	0.02	1.02	1.38*E* − 04	0.02	1.02	5.08*E* − 03
AC093627.3	0.12	1.13	2.66*E* − 04	0.16	1.18	1.35*E* − 05
DDN-AS1	0.35	1.42	1.95*E* − 06	0.22	1.25	4.22*E* − 03
GNAS-AS1	0.46	1.58	7.49*E* − 03	0.68	1.98	7.02*E* − 04
AC011442.1	0.39	1.48	1.54*E* − 02	0.72	2.06	3.22*E* − 03

**Table 2:** The comparative analysis of the risk score with other clinical factors in univariable and multivariable Cox regression models.

Features	Univariable Cox regression				Multivariable Cox regression			
	*P* value	HR	Lower 95% CI	Upper 95% CI	*P* value	HR	Lower 95% CI	Upper 95% CI
Risk score	6.82*E* − 12	19.7	8.41	46.2	7.33*E* − 12	19.7	8.41	46.4
Gender (female/male)	0.30	0.68	0.33	1.41	0.22	0.60	0.27	1.35
Race (white/other)	0.23	0.64	0.30	1.34	0.47	0.75	0.35	1.64
Age	0.82	1	1	1	0.72	1	1	1

## Data Availability

Previously reported gene expression and clinical data were used to support this study and are available at TARGET (Therapeutically Applicable Research To Generate Effective Treatments) database (https://ocg.cancer.gov/programs/target/data-matrix). These prior studies (and datasets) are cited at relevant places within the text as references [14].

## Supplementary Materials

Supplementary Materials Supplementary Table S1: the 50 prognostic long noncoding RNAs and statistical significance (*P* value) in osteosarcoma.
